# Achieving Open Access to Conservation Science

**DOI:** 10.1111/cobi.12346

**Published:** 2014-08-27

**Authors:** Richard A Fuller, Jasmine R Lee, James E M Watson

**Affiliations:** * School of Biological Sciences, University of QueenslandBrisbane, QLD 4072, Australia; † School of Geography, Planning and Environmental Management, University of QueenslandBrisbane, QLD 4072, Australia; ‡ Global Conservation Program, Wildlife Conservation Society2300 Southern Boulevard, Bronx, New York 10460, U.S.A.

**Keywords:** communications, ethics, funding, governance, philanthropy, policy, comunicación, ética, filantropía, financiamiento, gobernanza, política

## Abstract

Conservation science is a crisis discipline in which the results of scientific enquiry must be made available quickly to those implementing management. We assessed the extent to which scientific research published since the year 2000 in 20 conservation science journals is publicly available. Of the 19,207 papers published, 1,667 (8.68%) are freely downloadable from an official repository. Moreover, only 938 papers (4.88%) meet the standard definition of open access in which material can be freely reused providing attribution to the authors is given. This compares poorly with a comparable set of 20 evolutionary biology journals, where 31.93% of papers are freely downloadable and 7.49% are open access. Seventeen of the 20 conservation journals offer an open access option, but fewer than 5% of the papers are available through open access. The cost of accessing the full body of conservation science runs into tens of thousands of dollars per year for institutional subscribers, and many conservation practitioners cannot access pay-per-view science through their workplace. However, important initiatives such as Research4Life are making science available to organizations in developing countries. We urge authors of conservation science to pay for open access on a per-article basis or to choose publication in open access journals, taking care to ensure the license allows reuse for any purpose providing attribution is given. Currently, it would cost $51 million to make all conservation science published since 2000 freely available by paying the open access fees currently levied to authors. Publishers of conservation journals might consider more cost effective models for open access and conservation-oriented organizations running journals could consider a broader range of options for open access to nonmembers such as sponsorship of open access via membership fees.

Obtención de Acceso Abierto a la Ciencia de la Conservación

**Resumen:**

La ciencia de la conservación es una disciplina de crisis en la que los resultados del cuestionamiento científico deben hacerse disponibles de manera rápida para quienes están implementando el manejo. Evaluamos la extensión a la cual está disponible para el público la investigación científica publicada desde el año 2000 en 20 revistas de ciencia de la conservación. De los 19, 207 artículos publicados, 1, 667 (8.68%) están libres para descargar de un repositorio oficial. Además, sólo 938 artículos (4.88%) cumplen con la definición estándar de acceso abierto en la cual el material puede reutilizarse libremente siempre y cuando se le dé atribución a los autores. Esto se compara pobremente con un conjunto comparable de 20 revistas de biología evolutiva, donde 31.93% de los artículos están libres para descargar y el 7.94% son de acceso abierto. Diecisiete de las 20 revistas de conservación ofrecen una opción de acceso abierto, pero menos del 5% de los artículos están disponibles por medio del acceso abierto. El costo de acceder al cuerpo completo de la ciencia de la conservación llega a estar entre los miles de dólares por año para suscriptores institucionales, y muchos practicantes de la conservación no pueden acceder a la ciencia de paga en sus lugares de trabajo. Sin embargo, iniciativas importantes como Research4Life están poniendo a la ciencia a disponibilidad de organizaciones en países en desarrollo. Urgimos a los autores de la ciencia de la conservación que paguen por acceso abierto en una base por artículo o que escojan publicar en revistas de acceso abierto, tomando en consideración asegurar que la licencia permita reutilizar siempre y cuando se proporcione atribución. Actualmente, costaría $51 millones hacer que toda la ciencia de la conservación publicada desde 2000 esté disponible libremente al pagar las cuotas de acceso abierto que actualmente impuestas a los autores. Los publicadores de revistas de la conservación pueden considerar modelos más rentables para el acceso abierto y las organizaciones orientadas a la conservación que administran revistas podrían considerar un campo más amplio de opciones de acceso abierto para quienes no son miembros, como el patrocinio de acceso abierto por medio de pagos de membrecía

## Introduction

Unlike the pure sciences, whose *raison d’être* is to discover how the world works, the applied environmental sciences attempt to influence how Earth's resources are managed by people. Nowhere is this more true than in conservation science, where the scale and rapidity of the biodiversity crisis has made effective translation of knowledge into action one of its most pressing goals (Soulé 1985; Robinson 2006; McKinley et al. 2012). Although there has been enormous growth in the output of conservation scientists in the past 20 years (Robinson 2006), there have been repeated criticisms that translation of scientific advances into on-the-ground action is too slow, the so-called implementation gap (Knight et al. 2008; Arlettaz et al. 2010; Matzek et al. 2014). There are many barriers to conservation science being effective on the ground (Sutherland et al. 2004) and many suggestions of how to overcome them (Shanley & Lopez 2009; Sunderland et al. 2009; Braunisch et al. 2012). For example, Jenkins and Maxwell (2011) argue that to be effective, conservation scientists must actively escort their recommendations through the implementation processes via any process possible (e.g., serving on advisory panels, writing submissions on policy documents, tailoring reports specifically for implementing agencies). Arlettaz et al. (2010) go a step further and argue that when there is no clear pathway to implementation, the onus is on conservation scientists themselves to implement their recommendations directly.

Despite our improvement in understanding the implementation gap in conservation science, one of the oldest barriers remains resolutely in place—the fact that much of the scientific literature is only accessible by subscription or pay-per-view download (Harnad et al. 2008; Taylor et al. 2008). In a world where budgets are tight, this paywall is restricting access to conservation science by individuals and organizations tasked with the challenge of managing Earth's resources and halting the extinction crisis. Of course, access to primary science is only one of many reasons for the implementation gap, but better access is at least one step the conservation science community can take to get its house in order. For example, surveying practitioners managing over 1000 protected areas in Australia, Cook et al. (2010) discovered that 60% of conservation management decisions rely on experience-based information, and many practitioners report having insufficient evidence to assess their management decisions. Similarly, only 23% of practitioners surveyed by Pullin et al. (2004) used published scientific papers as a source for decision making. Of those that never accessed the literature, 65% identified the key barrier being that primary literature is too time consuming to locate and access (Pullin et al. 2004). A recent study by Walsh et al. (2014) found that conservation practitioners were likely to change nearly half of their proposed management actions after having access to a summary of the primary literature on a topic. As a first step to bridging the implementation gap it is critical that scientific evidence be made available to inform the decisions of those actually undertaking conservation action.

Science proceeds incrementally, with new knowledge being built upon the foundations of previous work. As such, it is critical that the results of previous researchers be available to those conducting new work, and a major industry has emerged around publishing and archiving the scientific literature (Taylor et al. 2008). However, this industry has historically focused on communicating the results of science to other scientists, and there has been little regard to public access. Recently, however, this has begun to change rapidly with the emergence of wholly open access journals (Laakso et al. 2011) and the option for authors to pay for free public access to individual articles (Solomon & Bjork 2012). Freely available journal articles are downloaded more frequently yet are not cited more frequently than subscription-only articles, hinting that free access could be especially important for people that primarily consume science rather than produce it (Davis et al. 2008; Davis 2011).

Although the recent trend toward open access is potentially good news for the rapid dissemination of conservation science, it is unclear to what extent this open access revolution has permeated conservation science. We measured how much of the conservation science literature produced since 2000 has been made universally and freely available by the publisher, and compare this with levels of open access in evolutionary biology, a related discipline, but one that is focused largely on discovering how the world works rather than trying to influence how it is managed.

## Methods

We began with the list of all 37 journals comprising the category Biodiversity and Conservation in the 2011 Thomson Reuters Journal Citation Report. To this list, we added 7 titles we consider core disciplinary journals within the field of conservation science (*Pacific Conservation Biology*, *Conservation and Society*, *Conservation Evidence*, *Current Opinion in Environmental Sustainability*, *Environmental Conservation*, *Insect Conservation and Diversity*, and *Journal of Insect Conservation*). Although many interdisciplinary journals publish important papers in conservation science, for the purpose of this exercise we wanted to measure rates of open access in those journals with a primary focus on conservation—these are the trade journals of the discipline and represent a set of journals committed primarily to publishing conservation science. We scanned the scope statements for each of these journals to select those with a primary focus on conservation science. We recognize that there are always difficult cases that complicate any classification. This process yielded a set of 20 conservation journals for analysis (Table1).

**Table 1:** Accessibility of articles published in major conservation science journals from 2000 to 2013

Journal	Date range	No. articles published	No. open access articles^a^	No. freely available articles^a^	Open access (%)	Author open access fee per article (US$)	Institutional subscription cost (US$)^b^	Publisher
*Conservation Evidence*	2004–2013	244	244	0	100	0	N/A	Cambridge Univ
*Natureza & Conservação*	2010–2013	91	91	0	100	0	N/A	ABECO
*Tropical Conservation Science*	2008–2013	187	187	0	100	0	N/A	Mongabay.com
*Conservation and Society*	2003–2013	247	215	32	87.04	0	N/A	Medknow
*Biological Invasions*	2000–2013	1839	58	12	3.15	3,000	831*	Springer
*Conservation Genetics*	2000–2013	1371	32	0	2.33	3,000	936‡	Springer
*Biodiversity and Conservation*	2000–2013	2635	52	11	1.97	3,000	3,771‡	Springer
*Journal of Insect Conservation*	2000–2013	685	11	19	1.61	3,000	1,497‡	Springer
*Pacific Conservation Biology*	2000–2013	374	4	0	1.07	N/A	362‡	Surrey Beatty
*Conservation Genetics Resources*	2009–2013	1003	10	26	1	3,000	616*	Springer
*Diversity and Distributions*	2000–2013	1050	10	115	0.95	3,000	8,620*	Wiley
*Conservation Biology*	2000–2013	2300	16	92	0.70	3,000	1,584*	Wiley
*Animal Conservation*	2000–2013	729	2	105	0.27	3,000	807*	Wiley
*Biological Conservation*	2000–2013	4160	5	64	0.12	2,500	4,304‡	Elsevier
*Oryx*	2000–2013	805	1	35	0.12	2,700	763*	Cambridge Univ Press
*Avian Conservation and Ecology*	2006–2013	99	0	99	0	730	N/A	SCO & BSC
*Conservation Letters*	2008–2013	273	0	40	0	3,000	376†	Wiley
*Environmental Conservation*	2000–2013	490	0	28	0	2,700	704*	Cambridge Univ Press
*Insect Conservation and Diversity*	2008–2013	240	0	51	0	3,000	1,430*	Wiley
*Journal for Nature Conservation*	2002–2013	385	0	0	0	3,000	318‡	Elsevier
**Total**		19207	938	729	4.88		26,919	

a Where more articles have been published than the sum of open access and freely available articles, the remaining articles are subscription access.

b Subscription cost represents the cheapest option available to a small institution. Type of subscription

* institution of any size, online only

† small institution, online only

‡ institution of any size, print and online.

We determined the level of accessibility for the research articles published in each journal by manually scanning every issue published between 1 January 2000 and 31 December 2013 or from the first issue of the journal if that postdated 2000. Although many journals began offering open access options after 2000, we used this common start date for all journals because we were interested in the pattern and prevalence of open access from the point of view of the science user rather than the reasons for the access itself; we included analysis of free availability, which can be conferred on papers of any age in a journal's archive; and the decision to offer an open access option is part of the drive to increase accessibility and hence part of the phenomenon we are trying to measure. Our search was conducted in January 2014.

We classified papers as open access, freely available, or subscription access and used these terms strictly as defined here throughout this paper. Our definition of *open access* was based on the Budapest Open Access Initiative (http://www.budapestopenaccessinitiative.org), which requires that an article may be freely re-used for any lawful purpose providing attribution is given to the authors. Open access is particularly important for conservation science because it, for example, allows work to be reused, repackaged, or repurposed by individuals or organizations (commercial or noncommercial) in a way that can explain its meaning to end users without having to seek permission or make payments to license holders. This is similar in spirit to a Creative Commons Attribution license (CCBY; http://creativecommons.org) currently offered to authors by many scientific journals. Creative Commons licenses form the basis of many journals' open access policies, and the CCBY website contains plain English summaries of the main restrictions associated with different kinds of licenses. If we were able to download a paper freely from the journal's official archive from a private computer not linked to a university network but it did not conform to our definition of open access, we classified it as freely available. Such papers either had additional restrictions attached to them (e.g., excluding commercial reuse or the production of derivatives) or retained all rights and had simply been made freely available online temporarily or permanently by the license holder. We classified all remaining articles as subscription access.

We did not include access to journal articles via pre-print servers because these do not represent the final published version of the manuscript and can be hard for nonspecialists to navigate, although it is worth noting that preprint servers such as arXiv.org are major repositories of information in several disciplines including physics and mathematics and could play a role in access to conservation science if conservation articles reached a critical mass in such repositories. Many journal websites have an index page for all volumes and issues; clicking on each issue reveals which papers are freely downloadable via a marker beside open access articles. For those journals that lacked visual markers, we clicked on each article to determine whether or not it was downloadable and then inspected the license conditions for any downloadable papers.

We experienced great difficulty in determining the precise license conditions associated with many freely downloadable papers, and despite extensive correspondence with publishers we remained unsure in some cases. We found many examples of publishers attempting to charge for reuse of papers marked by the journal as open access (e.g., via the Rightslink permissions facility used by Wiley and Elsevier), and where this was the case and there was no clear license information to the contrary on the published version of the paper, we assumed that these papers did not meet our strict open access definition. However, if an open access license was clearly stated on the published version of the paper, we considered it open access even if a charge was being levied because correspondence with publishers suggested some charges were being requested in error during the period of our data collection. In other cases, the published version of the paper contained links to generic information about a range of license options offered by the publisher that did not specify the precise conditions associated with that paper. Thus, when classifying the papers, we considered them open access only if there was clear evidence that a paper conformed to our definition. When we were in doubt, we classified papers as freely available. Subscription access articles were much easier to identify with confidence because they were simply not downloadable from a private computer.

We considered only original research articles in our count. This included research articles, brief communications, literature reviews, essays, and research or technical notes. Letters, book reviews, commentaries, responses, obituaries, editorials, and errata were not included, although variability in the terminology used for individual items meant that some case-by-case decisions were needed to determine whether an item represented a piece of scientific research. When we remained uncertain, we counted it as a research article if it had an abstract. We repeated this process for a comparable set of 20 evolutionary biology journals (*The American Naturalist*, *Biological Journal of the Linnean Society*, *BMC Evolutionary Biology*, *Development Genes and Evolution*, *Evolutionary Applications*, *Evolutionary Bioinformatics*, *Evolutionary Biology*, *Evolutionary Ecology*, *Evolutionary Ecology Research*, *Evolution*, *Genome Biology and Evolution*, *Heredity*, *Insect Systematics & Evolution*, *Journal of Evolutionary Biology*, *Journal of Human Evolution*, *Journal of Molecular Evolution*, *Molecular Biology and Evolution*, *Molecular Ecology*, *Molecular Phylogenetics and Evolution*, *Plant Systematics and Evolution*). We also recorded the cost of downloading each journal article for a nonsubscriber and scaled this up by the number of articles that were not open access per journal to estimate the total cost in U.S. dollars of accessing the literature on an ad hoc basis. We also calculated the cost to a small institution of annual subscriptions by collating information on the cheapest available option to access the literature published in the 15 conservation journals that were not freely available.

## Accessibility of conservation science literature

Conservation scientists have been busy in the past decade; 19,207 papers were published in the 20 core conservation journals since 2000 (Table1). Of these, 938 (4.88%) are open access, 729 (3.80%) were freely available in January 2014, and the remaining 91.3% of recent conservation science required some form of subscription or pay-per-view to be accessed. In the 20 core evolutionary biology journals, 36,093 papers were published in the same period, 2,705 (7.49%) of which are open access, 8,820 (24.4%) were freely available in January 2014, and the remaining 68.7% were subscription access. Five conservation journals that make all of their content freely available have been established since 2000, but correspondence with the journals confirmed that only 4 of these conform to our strict definition of open access (*Conservation Evidence; Natureza & Conservação; Tropical Conservation Science; Conservation and Society)*. Among the remaining 15 journals, open access rates were very low, ranging from 3.15% (*Biological Invasions*) to 0.12% (*Oryx*); 16 journals had an open access rate below 5% (Table1).

**Figure 1:**
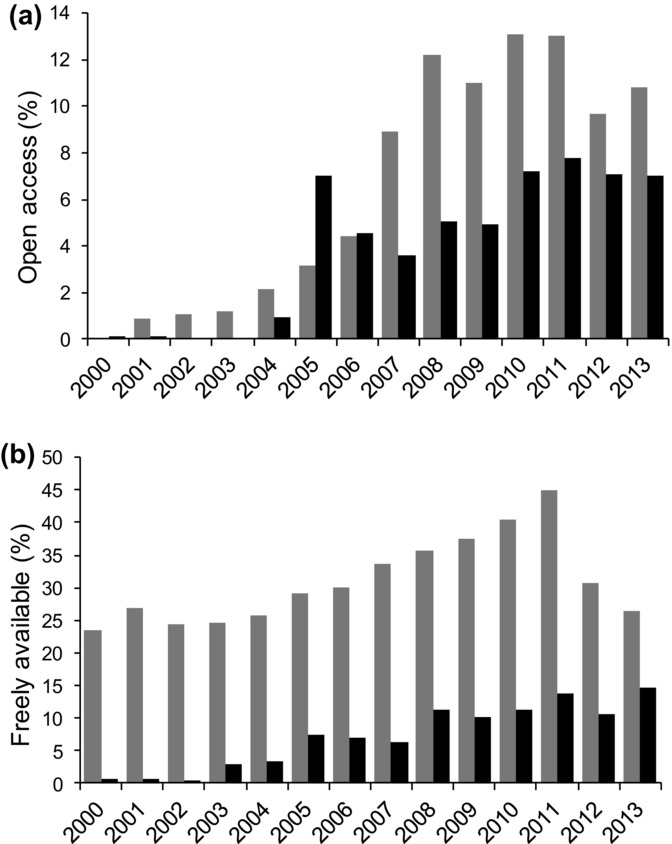
Temporal change in research articles in conservation science (black) and evolutionary biology (gray) (a) published as open access and (b) made freely available from 2000 through 2013.

Evolutionary biology research has achieved relatively high rates of open access and in particular free availability since 2000; there has been a period of stability since about 2007 (Fig.1; Supporting Information). Free availability of evolutionary biology articles is lower in the most recent few years, which is consistent with the lagged embargo (i.e., a period after which content is made freely available) commonplace in that discipline (Fig.1b). In contrast, open access to conservation science grew rapidly from a near-zero rate of 0.15% of articles published in 2000 to 7.0% of papers published in 2013, the last full year of data collection (Fig.1a). There has also been steady growth in free availability (Fig.1b). The 4 conservation journals with the highest open access rate were all established part way through the study period (Table1), suggesting that it has been more difficult for established journals to increase open access rates than for new journals to begin with an open access model. Indeed, access to science published in the 11 established conservation science journals (i.e., those issuing volumes throughout the study period) was much lower than the overall mean across all journals; 191 of 16,438 articles (1.17%) published in these journals are open access.

From a science user's perspective, the cost to download individual articles in the conservation science literature ranged from $31.50 to $45. Purchasing one-time access to all currently closed articles would cost $679,099. Subscriptions to online versions of all the conservation journals we considered that do not currently provide open access cost $26,919 per year for a small institution (Table1). From a science producer's perspective, the cost to authors for making a conservation science article open access or freely available, as distinct from publication costs, ranged from zero (*Conservation and Society*, *Conservation Evidence*, *Natureza & Conservação*, *Tropical Conservation Science*) to $3000 (11 journals). On this basis, it would cost $51,258,370 to pay the necessary fees to make all remaining conservation science published since 2000 open access under the current publication model and an annual average of $5,088,993 to cover the cost of open access fees for the science produced in the last 3 years (2011–2013).

## Improving access to conservation science

Open access to conservation science is substantially lower than evolutionary biology, and free availability is less than a third. This is unfortunate and ironic, given that conservation science is an applied science with an urgent deadline (Martinich et al. 2006). Although open access has grown in conservation science, this has largely been driven by the appearance of wholly open access journals rather than improved access to existing journals. The lack of uptake of open access by the traditional journals is surprising considering that many of the main publishers of these conservation journals are witnessing the growth of open access in evolutionary biology journals, although it is noteworthy that publishers achieving the highest rates of open access in conservation science are also those achieving high rates in evolutionary biology (Table1 & Supporting Information). The traditional journals remain the most prestigious places for conservation science to be published, and given the high esteem in which they are regarded within the scientific community, it is unfortunate that public access to the science they contain is so narrowly restricted. It seems at least plausible that authors are less motivated to pay for open access when publishing in a journal they perceive to have a wide circulation.

When the discipline of conservation biology was being first formed, Soulé (1986) emphasized that communication between science and practice had to improve. Although there have been many remarkable conservation successes over the past 3 decades (Robinson 2006), it is also apparent that many conservation projects are struggling to arrest biodiversity loss (Balmford & Cowling 2006; Hayes 2006), and conservation biology remains a crisis discipline (Redford & Taber 2000). One of the reasons for this failure is the fact that science is not always informing conservation practice, the so-called research-implementation gap (Pullin et al. 2004; Knight et al. 2008; Sunderland et al. 2009). There are many reasons for the persistence of the research–implementation gap, including the complexity of factors affecting on-the-ground decisions (Cook et al. 2012), variable scientific literacy of field practitioners (Pullin et al. 2004), poor access to the literature for those from developing countries (Karlsson et al. 2007; Taylor et al. 2008), a failure to embrace interdisciplinarity by conservation scientists (Campbell 2001), and by implication a lack of engagement by conservation scientists in the economic and policy sectors (Czech 2006). However, common among critiques is the lack of access to published literature, suggesting that a high proportion of papers published in scientific journals by conservation biologists are seldom read outside of the academic world. Although not all practitioners need or desire to read the primary literature, very few conservation organizations are without one or more staff members who are tasked with accessing, learning from, or producing primary science relevant to the organization's mission.

Dissemination of peer-reviewed research ensures that research communities are able to build on and share existing knowledge with practitioners and policy makers, highlight important new discoveries, and avoid duplicating failed efforts in either research or implementation. Scientific journals play a central role in this and are the principal medium for disseminating research results across the global scientific community. However, there is ample evidence that access to scientific journals is highly divided between developed and developing countries (Godlee et al. 2000; Taylor et al. 2008). In developing countries, where implementation of conservation science will have the most biodiversity impact (Lee & Jetz 2008), weak institutional infrastructures and governance, declining operational budgets, and currency weaknesses have resulted in institutions and libraries being unable to maintain subscriptions to the few journals they can afford (Sunderland et al. 2009). However, substantial progress is being made in this area, for example, Research4Life grants free or discounted access to scientific content from many of the world's biggest journal publishers to agencies in developing countries (http://www.research4life.org).

Our results are a stark warning that despite the growth of open access across science in general, dissemination through open access remains extremely small for the majority of publishing conservation scientists. One reason for this inertia is perhaps that even with rigorous peer review, new electronic journals cannot rapidly attain the same status as traditionally printed journals. However, there are some fine examples of the successful emergence of new journals that have already achieved considerable prestige such as *PLoS ONE*, indicating that this need not always be the case. There are also clear indications that governments and institutions are becoming increasingly motivated to make the results of publicly funded research available to the taxpayers who funded it (Harnad et al. 2008; Calver & Bradley 2010; Finch 2012).

We see 2 simple ways in which access to conservation science could be radically improved in the short term. Most immediately, we as conservation scientists could begin placing more emphasis on ensuring our work is made open access, either by choosing to publish in wholly open access journals or paying the open access fee as a matter of course for new papers. Of course, some grant giving bodies do not permit recipients to use funds to pay open access fees, the applied nature of many conservation science projects might deter applicants from budgeting for such fees in grant proposals, and conservation grants often have small budgets. Journals could also consider marketing their open access options much more vigorously to authors, who would in most cases perhaps be more prepared to pay for open access rather than obscure and usually optional page charges for which there is no tangible return. Ensuring our conservation science is open access will maximize its chance for real world impact, something that personally motivates many conservation scientists, and is becoming increasingly important in research assessment exercises around the world. Metrics that report on how many papers each conservation scientist has made publicly available could be produced and sit alongside an author's H-index and other bibliometrics such as Altmetric, which provides a score for published articles based on the level of online discussion and sharing (http://www.altmetric.com).

There are many researchers who cannot afford open access fees, and they should not be disadvantaged. In these cases, sponsorship from conservation organizations, benefactors, or the publishers themselves could be appropriate. Indeed, research councils are beginning to require open access publication or preprint posting (e.g., Research Councils UK, Finch 2012; National Institutes of Health, http://www.nih.gov). Professional societies that run journals, such as the Society for Conservation Biology, could play an important role in this, for example, by including options to sponsor open access in membership fees. This is undoubtedly a delicate area for those societies who rely heavily on the revenue stream from their journals, but the impact of taking bold decisions might not be as dramatic as one might expect. For example, the New Zealand Ecological Society funded a transition of their flagship journal (the *New Zealand Journal of Ecology*) to open access, and there has been no perceptible negative consequence for membership so far (J. Monks, personal communication).

A second (and not necessarily mutually exclusive) avenue for improving open access is to negotiate lagged free access to the conservation science journals. Publishers profit from a journal largely because of its reputation for quality, and professional subscriptions are primarily driven by those who need access to the current science delivered in the most recent issues. So, although there is clearly a commercial benefit to having this latest science behind a paywall, there is perhaps a lesser business imperative in constraining older science in the same way. Indeed, our data show that lifting the paywall after 2 years contributes enormously to making evolutionary biology freely available (Fig.1b), and content in all British Ecological Society journals published since 1998 are freely available as are more current articles 2 years after publication. This kind of model seems a relatively easy way to significantly increase access to science without asking the authors to pay and without unduly affecting journals’ balance sheets. However, most current science would remain locked up, and a lagged embargo might reduce the rate of library subscriptions or authors paying for temporary open access. This said, lagged open access combined with comprehensive access to preprints of recent papers could represent a large step forward in access to conservation science.

Whatever the solution to improving access to conservation science, it is clear that we must take immediate action to make our science more accessible and therefore more likely to be used to solve real world conservation problems. Obviously the issue of open access is but one cog in a larger machine that turns conservation science into conservation action, but it is time to get our house in order within the field of conservation science. Conservation is one of the few scientific disciplines that depend on practitioners for success. It makes sense to provide those who undertake the practice of conservation access to everything we know.

## Supporting Information

A table showing access to articles published in major evolutionary biology journals between 2000 and 2013 (Appendix S1) is available online. The authors are solely responsible for the content and functionality of these materials. Queries other than absence of the material should be directed to the corresponding author.

Appendix S1 Access to articles published in major evolutionary biology journals between 2000 and 2013. Where more articles have been published than the sum of open access and freely available articles, the remaining articles are subscription access.
