# Supplementary material for: Achieving Open Access to Conservation Science
Source: Conserv Biol. 2014 Aug 27;28(6):1550–7. doi: 10.1111/cobi.12346 (PMC4241051; doi:10.1111/cobi.12346)
Supplement: Appendix S1 — Access to articles published in major evolutionary biology journals between 2000 and 2013. Where more articles have been published than the sum of open access and freely available articles, the remaining articles are subscription access. [file cobi0028-1550-SD1.docx]

**Appendix S1** Access to articles published in major evolutionary biology journals between 2000 and 2013. Where more articles have been published than the sum of open access and freely available articles, the remaining articles are subscription access.

| Journal | Date range | # Articles published | # Open  access articles | # Freely  available articles | % Open access | Author open access fee per article (US$) | Publisher |
| --- | --- | --- | --- | --- | --- | --- | --- |
| *BMC Evolutionary Biology* | 2001-2013 | 2,335 | 2335 | 0 | 100 | 1,985 | BioMed Central |
| *Evolutionary Applications* | 2008-2013 | 360 | 105 | 255 | 29.17 | 1,950 | Wiley |
| *Evolutionary Biology* | 2007-2013 | 223 | 18 | 1 | 8.07 | 3,000 | Springer |
| *Development Genes and Evolution* | 2000-2013 | 881 | 36 | 3 | 4.09 | 3,000 | Springer |
| *Journal of Molecular Evolution* | 2000-2013 | 1,675 | 53 | 7 | 3.16 | 3,000 | Springer |
| *Plant Systematics and Evolution* | 2000-2013 | 1,662 | 51 | 0 | 3.07 | 3,000 | Springer |
| *Evolutionary Ecology* | 2000-2013 | 800 | 15 | 0 | 1.88 | 3,000 | Springer |
| *Evolution* | 2000-2013 | 3,618 | 29 | 117 | 0.80 | 3,000 | Wiley |
| *Journal of Evolutionary Biology* | 2000-2013 | 2,581 | 20 | 2153 | 0.77 | 3,000 | Wiley |
| *Molecular Ecology* | 2000-2013 | 4,891 | 32 | 229 | 0.65 | 3,000 | Wiley |
| *Biological Journal of the Linnean Society* | 2000-2013 | 2,340 | 7 | 74 | 0.30 | 3,000 | Wiley |
| *Molecular Biology and Evolution* | 2000-2013 | 3,473 | 3 | 3217 | 0.09 | 2,500 | Oxford Univ Press |
| *Molecular Phylogenetics and Evolution* | 2000-2013 | 3,875 | 1 | 28 | 0.03 | 3,000 | Elsevier |
| *American Naturalist* | 2000-2013 | 2,258 | 0 | 290 | 0 | 1,400 | Univ Chicago Press |
| *Evolutionary Bioinformatics* | 2005-2013 | 207 | 0 | 207 | 0 | 1,980 | Libertas Academica |
| *Evolutionary Ecology Research* | 2000-2013 | 1,035 | 0 | 9 | 0 | N/A | Evolutionary Ecology Ltd |
| *Genome Biology and Evolution* | 2009-2013 | 531 | 0 | 531 | 0 | 1,450 | Oxford Univ Press |
| *Heredity* | 2000-2013 | 1,797 | 0 | 1684 | 0 | 3,200 | Nature Publishing Group |
| *Insect Systematics & Evolution* | 2000-2013 | 349 | 0 | 7 | 0 | 2,800 | Brill |
| *Journal of Human Evolution* | 2000-2013 | 1,202 | 0 | 8 | 0 | 3,000 | Elsevier |
| **Total** |  | 36,093 | 2,705 | 8,820 | 7.49 |  |  |
